# Exercise Inhibits the Effects of Smoke-Induced COPD Involving Modulation of STAT3

**DOI:** 10.1155/2017/6572714

**Published:** 2017-10-18

**Authors:** Maysa Alves Rodrigues Brandao-Rangel, Andre Luis Lacerda Bachi, Manoel Carneiro Oliveira-Junior, Asghar Abbasi, Adriano Silva-Renno, Auriléia Aparecida de Brito, Ana Paula Ligeiro de Oliveira, Alessandra Choqueta Toledo-Arruda, Maria Gabriela Belvisi, Rodolfo Paula Vieira

**Affiliations:** ^1^Nove de Julho University, São Paulo, SP, Brazil; ^2^Brazilian Institute of Teaching and Research in Pulmonary and Exercise Immunology (IBEPIPE), School of Medical Sciences of São José dos Campos Humanitas and Universidade Brasil, São Paulo, SP, Brazil; ^3^Institute of Physical Activity Sciences and Sports, Post-Graduate Program in Human Movement Sciences, Cruzeiro do Sul University, São Paulo, SP, Brazil; ^4^Institute for Memory Impairments and Neurological Disorders (MIND Institute), University of California, Irvine, CA, USA; ^5^Laboratory of Experimental Therapeutics (LIM 20), Department of Medicine, School of Medicine, University of Sao Paulo, São Paulo, SP, Brazil; ^6^Respiratory Pharmacology Group, Airway Disease, National Heart and Lung Institute, Imperial College London, London, UK

## Abstract

**Purpose:**

Evaluate the participation of STAT3 in the effects of aerobic exercise (AE) in a model of smoke-induced COPD.

**Methods:**

C57Bl/6 male mice were divided into control, Exe, COPD, and COPD+Exe groups. Smoke were administered during 90 days. Treadmill aerobic training begun on day 61 until day 90. Pulmonary inflammation, systemic inflammation, the level of lung emphysema, and the airway remodeling were evaluated. Analysis of integral and phosphorylated expression of STAT3 by airway epithelial cells, peribronchial leukocytes, and parenchymal leukocytes was performed.

**Results:**

AE inhibited smoke-induced accumulation of total cells (*p* < 0.001), lymphocytes (*p* < 0.001), and neutrophils (*p* < 0.001) in BAL, as well as BAL levels of IL-1*β* (*p* < 0.001), CXCL1 (*p* < 0.001), IL-17 (*p* < 0.001), and TNF-*α* (*p* < 0.05), while increased the levels of IL-10 (*p* < 0.001). AE also inhibited smoke-induced increases in total leukocytes (*p* < 0.001), neutrophils (*p* < 0.05), lymphocytes (*p* < 0.001), and monocytes (*p* < 0.01) in blood, as well as serum levels of IL-1*β* (*p* < 0.01), CXCL1 (*p* < 0.01), IL-17 (*p* < 0.05), and TNF-*α* (*p* < 0.01), while increased the levels of IL-10 (*p* < 0.001). AE reduced smoke-induced emphysema (*p* < 0.001) and collagen fiber accumulation in the airways (*p* < 0.001). AE reduced smoke-induced STAT3 and phospho-STAT3 expression in airway epithelial cells (*p* < 0.001), peribronchial leukocytes (*p* < 0.001), and parenchymal leukocytes (*p* < 0.001).

**Conclusions:**

AE reduces smoke-induced COPD phenotype involving STAT3.

## 1. Introduction

Chronic obstructive pulmonary disease (COPD) is the leading respiratory disease and the fourth cause of death worldwide [1]. COPD is a common, preventable, treatable, and progressive multifactorial syndrome clinically characterized by persistent respiratory symptoms, such as dyspnea, cough, and sputum production [1]. The main risk factor for the development of COPD is tobacco smoking, which leads to pulmonary inflammation triggering pulmonary structural alterations, such as pulmonary remodeling and alveolar destruction, characterizing lung emphysema [1]. Although pulmonary inflammation presents a key role in COPD pathophysiology, systemic inflammation is also present in COPD and it is tough to be especially related to COPD exacerbations, impairment of cardiovascular response, metabolism, atrophy, and dysfunction of skeletal muscle [1, 2]. The underlying mechanisms involved in these responses are target by increased number of studies, and more recently, the involvement of signal transducers and activators of transcription pathway (STAT), as demonstrated by increased expression of STAT proteins, was described in lung tissue biopsies of COPD patients, specifically in airway epithelial cells and in parenchymal leukocytes [3].

Clinical studies demonstrate that aerobic exercise (AE) improves COPD management, reducing symptoms, exacerbations, and decline of the lung function, while improve quality of life [2, 4]. As part of the mechanisms involved in such response, experimental studies have demonstrated that AE reduces pulmonary oxidative stress and pulmonary inflammation, but to date, no study has investigated the effects of AE on systemic inflammation and on inflammatory transcription factors, such as STATs in models of COPD [5, 6].

Therefore, the present study investigated the effects of AE on pulmonary inflammation and emphysema, on systemic inflammation, and on STAT3 signaling in a smoke model of COPD in mice.

## 2. Material and Methods

All experiments were conducted according to Helsinki Convention for use and care of animals published in 2013 and were approved by the ethical committee of University of Sao Paulo (001/14).

### 2.1. Experimental Design

Sixty-four male C57Bl/6 mice (18–25 g) were obtained from Central Animal Facility of University of Sao Paulo, kept in pathogen-free conditions, and distributed (*n* = 2 × 8 mice per group) in the control, exercise (Exe), COPD, and COPD+Exe groups.

### 2.2. Experimental Model of Smoke-Induced COPD in Mice

The COPD and COPD+Exe groups were exposed twice a day to tobacco smoking (2 × 7 cigarette/day; 0.8 mg nicotine, 10 mg tar, 10 mg carbon monoxide/cigarette) in an acrylic box (28 liters), for 30 minutes per session, as adapted from Toledo et al. [5]. In summary, our experimental model added one more tobacco smoking exposition per day (in the afternoons), resulting in a carbon monoxide (CO) concentrations ranging between 250 and 350 ppm into the acrylic box and in a blood concentration of carboxyhemoglobin ranging between 10% ± 1.4%. The control and Exe groups were exposed only to air. This change resulted in an improved experimental model of COPD in contrast to Toledo's protocol, since our model induced more significant airway inflammation and emphysema. Therefore, smoke or air exposure was performed for initially 60 days (for the establishment of airway inflammation and emphysema, which are the main characteristics of COPD), followed by aerobic treadmill training and smoke or air exposure for more 30 days.

### 2.3. Treadmill Aerobic Test and Training

All mice were initially adapted to treadmill (25° incline, 3 days, 15 minutes/session, 0.2 Km/h) on days 57, 58, and 59 of the experimental protocol. On day 60 of the experimental protocol, treadmill test was performed in order to determinate the initial maximal exercise capacity, corresponding to the maximal velocity reached in the initial test as previously described [5]. Treadmill aerobic training begun on day 61 of the experimental protocol and was performed at 50% of maximal velocity reached in the initial test (25° incline, 4 weeks, 5 days/week, 60 minutes/session) [5].

### 2.4. Evaluation of the Lung Inflammation

Lung inflammation was evaluated through the analysis of the cellular content of total and differential leukocytes in the bronchoalveolar lavage (BAL) and by the analysis of cytokine levels in BAL. Following blood collection, mice were tracheotomized and cannulated, and the lungs were washed with 3 × 500 mL of phosphate-buffered saline (PBS) [7]. The recovered volume was centrifuged at 900*g*, 4°C, 7 minutes, and the supernatant was collected and stored at −86°C for cytokine analysis. Cell pellet was resuspended in 1000 *μ*L of PBS, and the total number of cells were counted using a hematocytometer (Neubauer Chamber, Karlsruhe, Germany) [7]. The differential cell count (neutrophils, lymphocytes, macrophages, and eosinophils) was performed through the cytospin preparations, in which slides were stained with Diff-Quick and 300 cells per slide were counted according to the hematological criteria [7].

The levels of IL-1*β*, IL-6, CXCL1, IL-10, IL-17, and TNF-*α* in BAL were analyzed by ELISA, using commercial kits from R&D Systems (MN, USA) and BioLegend (CA, USA) according to the manufacturer's recommendations [8].

### 2.5. Evaluation of the Systemic Inflammation

Under anesthesia (ketamine 10 mg/kg^−1^ and xylazine 100 mg/kg^−1^), laparotomy was performed and 1 mL of blood was collected in cava vein. By using approximately 20 *μ*L of blood, the whole blood cell analysis was performed using the hematology analyzer Sysmex XS-800i (Sysmex Europe GmbH, Norderstedt, Germany). The reminiscent blood volume was centrifuged at 900*g* during 7 minutes at 4°C, and the serum was stored at −86°C for cytokine (IL-1*β*, CXCL1, IL-10, IL-17, and TNF-*α*) measurements by ELISA using commercial kits from R&D Systems (MN, USA) and BioLegend (CA, USA) according to the manufacturer's recommendations [8].

### 2.6. Evaluation of Lung Emphysema and Airway Remodeling

After blood and BAL collection, the lungs were carefully removed, perfused and fixed using 4% formalin for 24 hours at a positive pressure (20 cm H_2_O), and submitted to histological routine. Five-micrometer slices were stained with hematoxylin and eosin for the analysis of the level of emphysema through the quantification of alveolar enlargement (Lm) [5]. Additional staining with picrosirius was performed to quantify collagen fiber deposition in the airway wall [5, 7]. In summary, five airways per mouse (all mice from all groups) were photographed by using an Olympus BX40 (Olympus, PA, USA) optical microscope and submitted to image analysis using the CellSens software (Olympus, PA, USA). The density of collagen fibers was measured using a standardized color threshold (corresponding to red staining) by CellSens software (Olympus, PA, USA) into the region between the basal membrane of epithelium until airway adventitia. The results were expressed as *μ*m^2^ of collagen fibers per *μ*m^2^ of tissue area [9].

### 2.7. Quantification of the Total and Phosphorylated STAT3 by Airway Epithelium, Peribronchial Leukocytes, and Parenchymal Leukocytes

Five-micrometer slices in slides pretreated with silane were submitted to immunohistochemistry routine, followed by incubation with the following primary antibodies: p-STAT3 (Tyr 705) (sc-7993) and STAT3 (C20) (sc-482) (Santa Cruz Biotechnology, Santa Cruz, CA, USA). The slides were incubated with proper secondary antibodies conjugated with biotin-streptavidin-peroxidase, followed by counterstaining with Harris hematoxylin [7–10]. The number of peribronchial leukocytes positive for each antibody was counted in two lung compartments: (i) in the peribronchial space (comprehended between the basal membrane and airway adventitia—which is relevant to understand the participation of STAT3 in bronchitis) [7, 9] and (ii) in the lung parenchyma (which is relevant to understand the participation of STAT3 in emphysema) [8]. In addition, the expression of p-STAT3 and STAT3 in the airway epithelium was quantified (which is relevant to understand the role of epithelium as a source of STAT3 in the pathophysiology of COPD) [9, 10]. The number of positive leukocytes was analyzed by combined image analysis with point-counting technique [7–9]. Counting was performed in five airways and in fifteen parenchymal fields for each animal of all experimental groups at 400x magnification. Stained peribronchial positive leukocytes were expressed as the number of positive cells per square millimeter [7–9], whereas airway epithelium protein expression was represented as a percentage of positive area of the epithelium [9, 10].

### 2.8. Statistical Analysis

The software GraphPad Prism 5.0 was used to perform the statistical analysis and graphs. All data were analyzed by one-way analysis of variance (ANOVA) followed by Bonferroni's test, since the analysis of the distribution of the data revealed normal distribution. The results were expressed as mean ± standard deviation. *p* values were considered significant at *p* < 0.05.

## 3. Results

### 3.1. Aerobic Exercise Inhibits Smoke-Induced Pulmonary Inflammation

**Figure 1:**
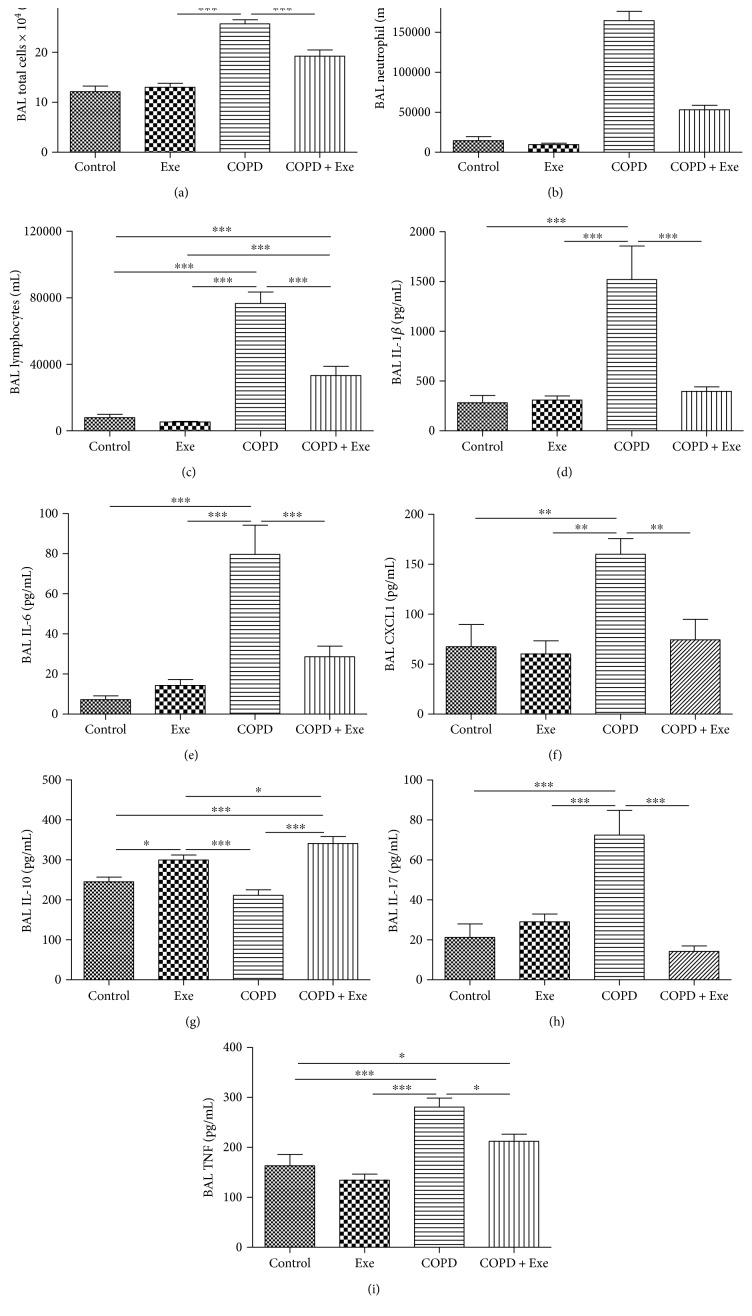
The results obtained in bronchoalveolar lavage (BAL) fluid of the 4 groups of mice: control, aerobic exercise (Exe), chronic obstructive pulmonary disease (COPD), and chronic obstructive pulmonary disease submitted to aerobic exercise (COPD+Exe). Number (×10^4^/mL) of (a) total cells, (b) neutrophils, and (c) lymphocytes. Concentration (pg/mL) of (d) IL-1*β*, (e) IL-6, (f) CXCL1, (g) IL-10, (h) IL-17, and (i) TNF-*α*. Data are presented as means and SD. ^∗^*p* < 0.05; ^∗∗^*p* < 0.01; ^∗∗∗^*p* < 0.001.

Immune cell influx and inflammatory mediators' levels were measured in the bronchial alveolar lavage (BAL) in all animals.  Figure 1 confirms the presence of lung inflammation in smoke-induced COPD. Chronic smoke exposure significantly increased the influx of leukocytes in BAL. This was represented by an increased total number of cells (*p* < 0.001), neutrophils (*p* < 0.001), and lymphocytes (*p* < 0.001) in the COPD group (Figures 1(a), 1(b), and 1(c)). Exercise administration, however, reduced the number of total cells (*p* < 0.001), neutrophils (*p* < 0.001), and lymphocytes (*p* < 0.001), in the COPD+Exe animals (Figures 1(a), 1(b), and 1(c)).

In addition, chronic smoke exposure significantly increased the BAL levels of inflammatory cytokines including IL-1*β* (*p* < 0.001), IL-6 (*p* < 0.001), CXCL1 (*p* < 0.001), IL-17 (*p* < 0.001), and TNF-*α* (*p* < 0.001), as well as decreased the BAL levels of anti-inflammatory cytokine IL-10 (*p* < 0.001) (Figures 1(d), 1(e), 1(f), 1(g), 1(h), and 1(i)). In contrast, exercise exposure significantly reduced the BAL levels of inflammatory cytokines IL-1*β*, IL-6, CXCL1, IL-17, and TNF-*α* in the COPD+Exe animals. Exercise, also, significantly increased the BAL levels of anti-inflammatory cytokine IL-10 in the COPD+Exe and Exe groups (Figures 1(d), 1(e), 1(f), 1(g), 1(h), and 1(i)).

### 3.2. Aerobic Exercise Inhibits Smoke-Induced Systemic Inflammation

**Figure 2:**
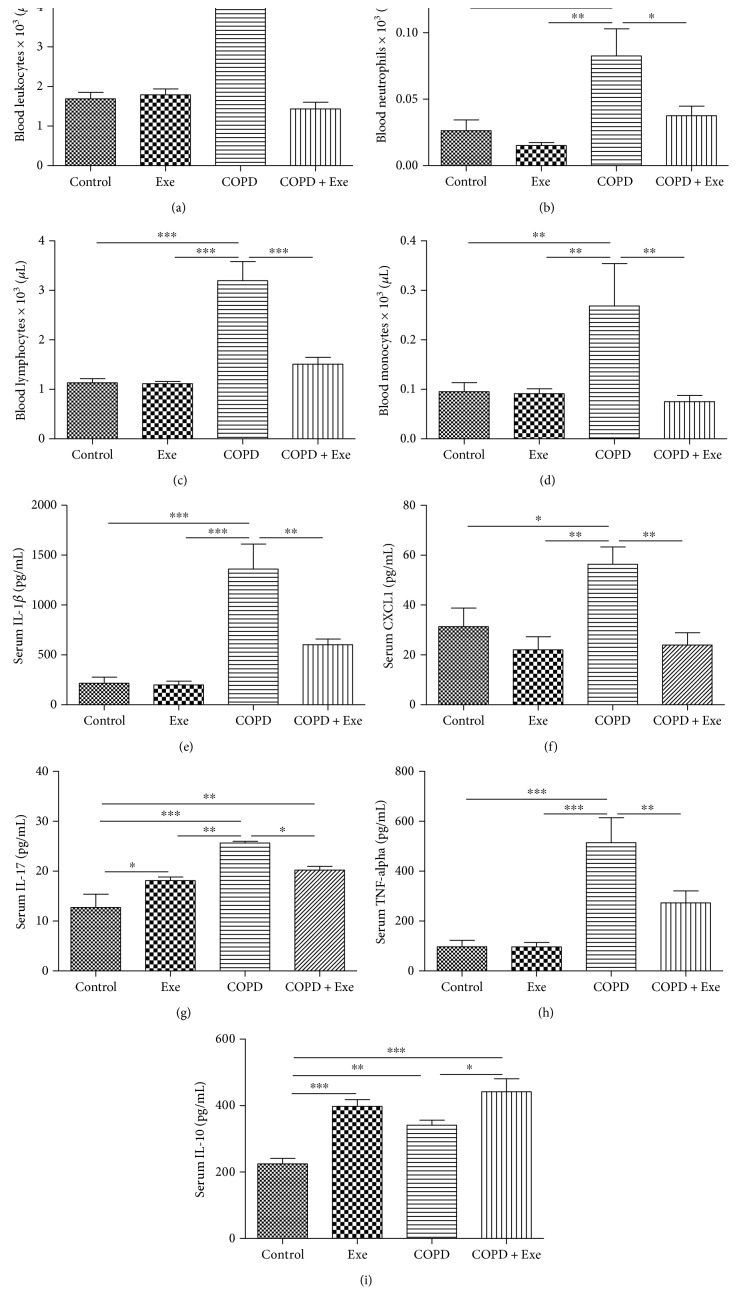
The results obtained in the blood and serum samples of the 4 groups of mice: control, aerobic exercise (Exe), chronic obstructive pulmonary disease (COPD), and chronic obstructive pulmonary disease submitted to aerobic exercise (COPD+Exe). Blood number (×10^3^/uL) of (a) leucocytes, (b) neutrophils, (c) lymphocytes, and (d) monocytes. Serum concentration (pg/mL) of (e) IL-1*β*, (f) CXCL1, (g) IL-17, (h) TNF-*α*, and (i) IL-10. Data are presented as means and SD. ^∗^*p* < 0.05; ^∗∗^*p* < 0.01; ^∗∗∗^*p* < 0.001.

To confirm that chronic smoking induces systemic inflammation, we evaluated the number of immune cells and the levels of inflammatory cytokines in the circulation of all animals. As shown in Figure 2, chronic smoke exposure significantly increased the number of total leukocytes (*p* < 0.001), neutrophils (*p* < 0.01), lymphocytes (*p* < 0.001), and monocytes (*p* < 0.01) in the COPD animals (Figures 2(a), 2(b), 2(c), and 2(d)). Similarly, smoke exposure increased serum levels of IL-1*β* (*p* < 0.001), CXCL1 (*p* < 0.01), IL-17 (*p* < 0.001), and TNF-*α* (*p* < 0.001) in the COPD animals (Figures 2(e), 2(f), 2(g), and 2(h)). In contrast, exercise administration significantly reduced the number of immune cells (Figures 2(a), 2(b), 2(c), and 2(d)) and the circulating levels of inflammatory markers (Figures 2(e), 2(f), 2(g), and 2(h)) in the COPD+Exe animals compared to the COPD group. Exercise administration also significantly increased the circulatory levels of IL-10 in the COPD+Exe and Exe groups compared to 2 other groups.

### 3.3. Aerobic Exercise Inhibits Smoke-Induced Airway Remodeling and Lung Emphysema

**Figure 3:**
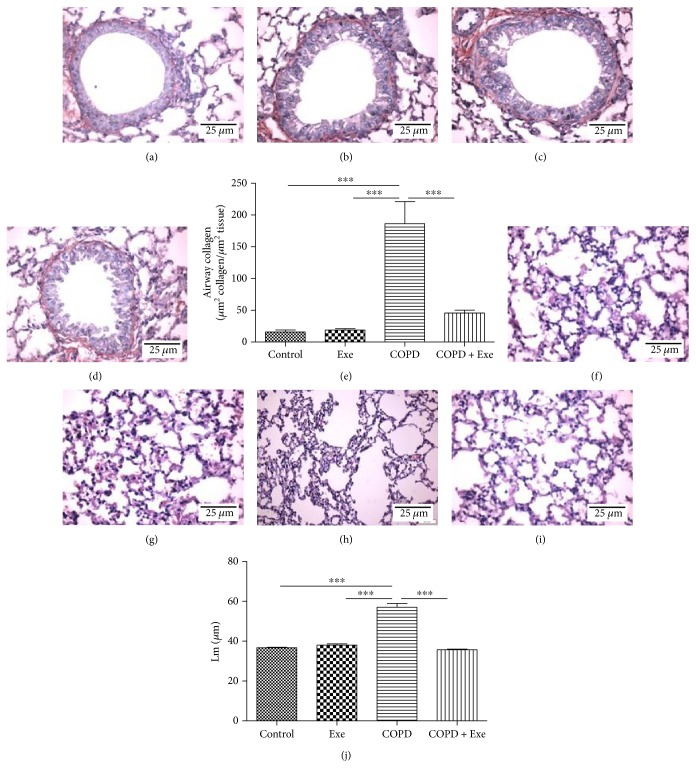
Representative photomicrographs of airways from the (a) control, (b) aerobic exercise (Exe), (c) chronic obstructive pulmonary disease (COPD), and (d) chronic obstructive pulmonary disease submitted to aerobic exercise (COPD+Exe) groups, stained with picrosirius (red staining). Scale bars: 25 mm. (e) Quantification of collagen fiber deposition in the airway wall (*μ*m^2^ collagen/*μ*m^2^ tissue) in the groups: control, exercise (Exe), chronic obstructive pulmonary disease (COPD), and chronic obstructive pulmonary disease submitted to aerobic exercise (COPD+Exe). Representative photomicrographs of hematoxylin and eosin-stained pulmonary parenchyma in the groups: (f) control, (g) exercise (Exe), (h) chronic obstructive pulmonary disease (COPD), and (i) chronic obstructive pulmonary disease submitted to aerobic exercise (COPD+Exe). Scale bars: 25 mm. (j) Quantification of alveolar enlargement (Lm, *μ*m). Data are presented as means and SD. ^∗∗∗^*p* < 0.001.

To confirm the establishment of structural pulmonary alterations induced by chronic smoke exposure, airway remodeling and lung emphysema were measured. Smoke exposure induced significant accumulation of collagen fibers in the airway wall of the COPD animals (Figures 3(a), 3(b), 3(c), 3(d), and 3(e)) compared to other groups. Similarly, chronic smoke exposure significantly increased lung emphysema in the COPD animals compared to other 3 groups (Figures 3(f), 3(g), 3(h), 3(i), and 3(j)). In contrast, exercise administration significantly reduced the accumulation of collagen fibers in airway wall in the COPD+Exe group (Figures 3(a), 3(b), 3(c), 3(d), and 3(e)). Likewise, exercise reduced lung emphysema in the COPD animals compared to the COPD group (Figures 3(f), 3(g), 3(h), 3(i), and 3(j)).

### 3.4. Aerobic Exercise Reduces Pulmonary STAT3 Expression and Activation

**Figure 4:**
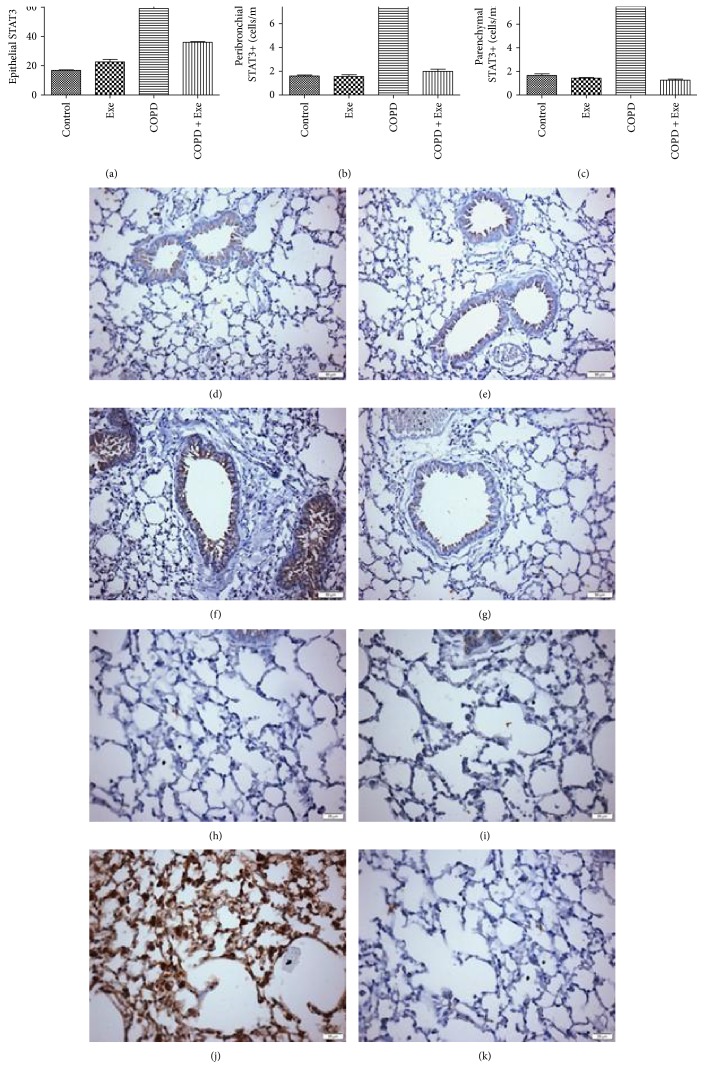
The results obtained in the evaluation of total STAT3 expression in airway epithelial cells (a), peribronchial leukocytes (b), and parenchymal leukocytes (c) in the groups: control, exercise (Exe), chronic obstructive pulmonary disease (COPD), and chronic obstructive pulmonary disease submitted to aerobic exercise (COPD+Exe). Representative photomicrographs of lung slices focusing on epithelial STAT3 expression from the (d) control, (e) exercise, (f) chronic obstructive pulmonary disease, and (g) chronic obstructive pulmonary disease submitted to aerobic exercise (COPD+Exe) groups and on parenchyma STAT3 expression from the (h) control, (i) exercise (Exe), (j) chronic obstructive pulmonary disease (COPD), and (k) chronic obstructive pulmonary disease submitted to aerobic exercise (COPD+Exe) groups. Scale bars: 25 mm. Data are presented as means and SD. ^∗^*p* < 0.05; ^∗∗∗^*p* < 0.001.

Figure 4 shows that chronic smoke exposure resulted in increased expression of STAT3 in three different lung compartments [airway epithelial cells (*p* < 0.001), peribronchial leukocytes (*p* < 0.001), and parenchymal leukocytes (*p* < 0.001)]. The results also demonstrated that exercise administration reduced STAT3 expression in all three lung compartments in the COPD+Exe group compared to the COPD group of animals (Figures 4(a), 4(b), and 4(c)).

**Figure 5:**
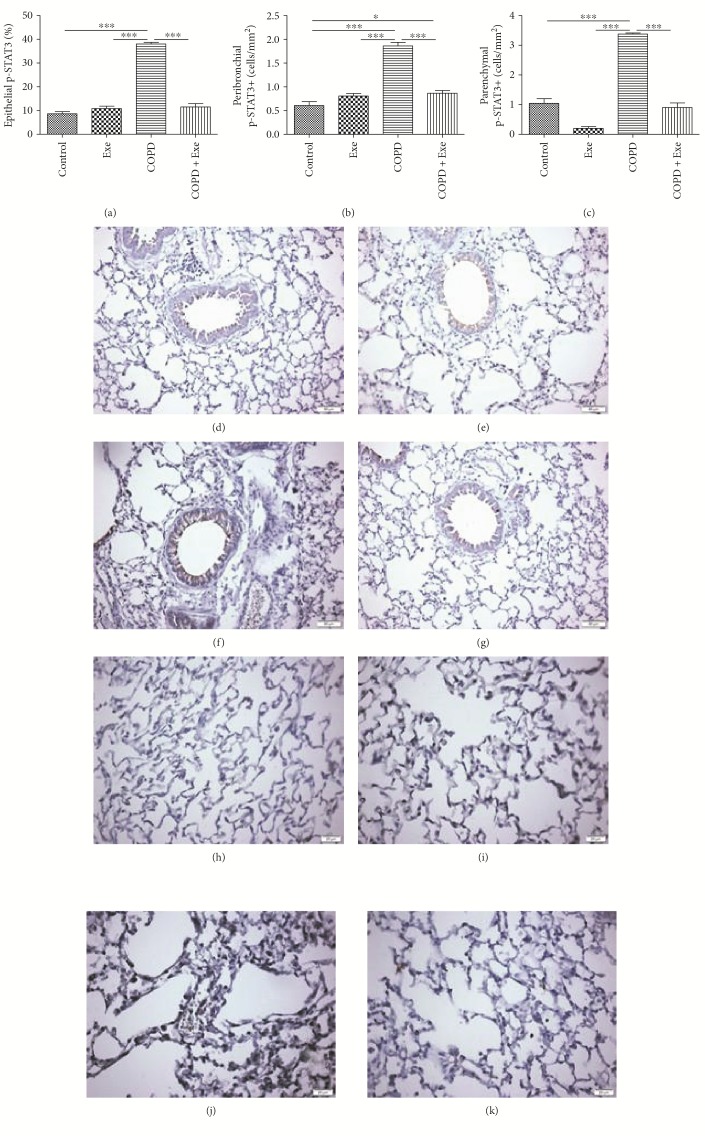
The results obtained in the evaluation of phosphorylated STAT3 (p-STAT3) expression in airway epithelial cells (a), peribronchial leukocytes (b), and parenchymal leukocytes (c) in the groups: control, exercise (Exe), chronic obstructive pulmonary disease (COPD), and chronic obstructive pulmonary disease submitted to aerobic exercise (COPD+Exe). Representative photomicrographs of lung slices focusing on epithelial p-STAT3 expression from the (d) control, (e) exercise, (f) chronic obstructive pulmonary disease, and (g) chronic obstructive pulmonary disease submitted to aerobic exercise (COPD+Exe) groups and on parenchyma p-STAT3 expression from the (h) control, (i) exercise (Exe), (j) chronic obstructive pulmonary disease (COPD), and (k) chronic obstructive pulmonary disease submitted to aerobic exercise (COPD+Exe) groups. Scale bars: 25 mm. Data are presented as means and SD. ^∗^*p* < 0.05; ^∗∗∗^*p* < 0.001.

Figure 5 shows that chronic smoke exposure resulted in increased pulmonary activation of STAT3, as demonstrated by increased expression of phosphorylated STAT3 (p-STAT3) in three different lung compartments airway epithelial cells (*p* < 0.001), peribronchial leukocytes (*p* < 0.001), and parenchymal leukocytes (*p* < 0.001) (Figures 5(a), 5(b), and 5(c)). Contrary to this, exercise significantly reduced expression of p-STAT3 in all three lung compartments in the COPD+Exe animals (Figures 5(a), 5(b), and 5(c)).

## 4. Discussion

The present study demonstrated that aerobic exercise attenuated lung inflammatory and remodeling responses and lung emphysema in a mouse model of COPD. In addition, although previous studies demonstrated that aerobic exercise is effective in reducing pulmonary COPD outcomes [4–10], the present study shows, for the first time, that aerobic exercise not only reduces pulmonary but also systemic COPD outcomes and this reduction may be associated to the inhibitory effects of aerobic exercise on STAT3 activation. More specifically, aerobic exercise lessened the proinflammatory response induced by smoke exposure, such as cellular infiltrate and proinflammatory cytokine levels in BAL and systemic circulation. In addition, aerobic exercise decreased both expression and phosphorylation of STAT3 in three different lung compartments, peribronchial leukocytes (which are relevant to underlying mechanisms of smoke-induced bronchitis), parenchymal leukocytes (which are relevant to underlying mechanisms of smoke-induced lung emphysema), and also by airway epithelial cells (which revealed to be an important source of STAT3 expression and activation in a model of smoke-induced COPD) [3].

It is widely accepted that cigarette smoke induces chronic pulmonary inflammation, which can affect not only the airways causing bronchitis but also the distal region of the lungs, contributing to the development of lung parenchyma [4, 11]. As a potent proinflammatory agent, cigarette smoke activates the production of different proinflammatory cytokines, such as IL-1*β*, IL-6, IL-8, IL-17, and TNF-*α*, which present a key role in both pulmonary inflammatory and structural alterations [12, 13]. These cytokines act attracting and activating leukocytes into the inflammatory site, leading to nonresolved inflammatory response, perpetuating the inflammatory process, especially for chronic respiratory diseases, such as COPD and asthma [12–15]. Regarding the pathogenesis of smoke-induced lung injury, studies demonstrated that the increased number of polymorphonuclear cells, as neutrophils, is a remarkable cell that must be considered [1, 13, 16, 17]. For instance, it has been demonstrated that in COPD, neutrophils are one of the predominant inflammatory leukocytes in the lung and contribute to airway remodeling as well as to the development of airway obstruction [1, 13, 16, 17]. In this context, our results revealed that the effectiveness of the model to induce chronic bronchitis is mainly characterized by increased number of neutrophils and lymphocytes in BAL, followed by increased levels of proinflammatory cytokines (IL-1*β*, IL-6, CXCL1, IL-17, and TNF-*α*). On the other side, aerobic exercise reduced all these inflammatory parameters, confirming the efficiency of aerobic exercise in reducing smoke-induced pulmonary inflammation. Exercise reducing neutrophil accumulation is particularly important for COPD, considering that neutrophils release several mediators that are involved not only with the inflammatory process but also with the airway remodeling, beyond to be related to a rapid decline in the forced expiratory capacity in the first second (FEV1) even in COPD patients using inhaled corticosteroids (ICS) [16, 17]. However, whether exercise-reduced neutrophils in a model of COPD could be related to an improved and sustained FEV1 in COPD patients is unknown, but is guaranteed for further clinical studies. Beyond neutrophils, lymphocytes also present an important role in COPD [18]. For instance, cytotoxic T cells (CD8+ T cells) are involved in emphysema development through the release of different cytotoxic substances, while CD4+ T cells release cytokines and chemokines increasing and perpetuating the inflammatory process [18]. In this way, the present study showed that chronic smoke exposure increased the number of lymphocytes in BAL, while aerobic exercise reduced it.

By the way, it is expected that the reduction of inflammation, especially in the lung, could minimize the COPD severity. Among several strategies that are been studied to prevent and treat the deleterious effects of chronic cigarette smoke exposition in the airways [19–21], the regular practice of physical exercise in moderate intensity has been highlighted [22]. According to the literature, exercise training presents an evident anti-inflammatory effect [23] and it leads to the improvement of both cardiopulmonary capacity [24] and pulmonary immune system in chronic respiratory diseases when performed in moderate intensity [5–11]. As part of the mechanism underlying the anti-inflammatory effects of exercise, during a session of physical exercise, activated skeletal muscle releases a myriad of molecules that act locally or in different tissues and organs, as adipose tissue, endothelium, the liver, brain, and lungs [22, 25, 26]. The most studied molecule released by skeletal muscle during physical exercise is IL-6 [27]. Exercise-induced IL-6 release from skeletal muscle is believed to precede the elevation of two well-known anti-inflammatory cytokines, IL-10 and IL-1ra [23, 27–29]. In the present study, we found increased concentration of both pulmonary and systemic IL-10 levels in the Exe and COPD+Exe groups. Furthermore, the classical anti-inflammatory effect of IL-10 could be denoted by the significant reduction in both pulmonary and systemic proinflammatory cytokines (IL-1b, IL-6, CXCL1, IL-17, and TNF-*α*) for the mice submitted to exercise (Exe and COPD+Exe groups).

Of note, the reduction of the proinflammatory molecule CXCL1, a potent chemoattractant of neutrophils [28–30], in BAL and in the serum of COPD+Exe group could putatively lead to the reduction of neutrophil migration and activation in airways and consequently minimize the damage of chronic exposition of cigarette smoke in the lung. Moreover, it was demonstrated that polymorphonuclear cell migration after a session of physical exercise is altered due to lack of response of cell-specific membrane receptors to specific chemokines, as well as to the alteration in the polarization of these cells [31]. The reduction of inflammatory cell migration in the lung can minimize the occurrence of the airway remodeling, which consequently favors the maintenance of pulmonary integrity and function [4, 5, 9]. Besides, studies have shown that physical exercise also acts in reducing the airway and parenchymal remodeling through lower elastic and collagen deposition, inhibition of mucus synthesis, and reduction of smooth muscle thickness [7–9]; effects were also observed in the present study using a smoke model of COPD. These important effects of aerobic exercise on airway remodeling and on lung emphysema, however, need further investigation regarding the mechanisms involved in such response as well as if similar response can also happen in COPD patients.

Several studies demonstrate that chronic exposure to cigarette smoke activates airway epithelium triggering different signaling pathways involved in the inflammatory and fibrotic responses associated to COPD development, such as signal transducer and activator of transcription (STAT) [3, 32], Janus tyrosine kinase (JAK) [3, 32], and the nuclear factor-*κ*B (NF-*κ*B) [33]. It has been reported in the literature that the increased concentration of the proinflammatory cytokine IL-6 can predict the development of COPD with higher sensitivity [33] and, in association with IL-8, can predict the mortality and morbidity in COPD patients [14]. Concerning pulmonary inflammation, it was observed that IL-6 directly activates STAT3 [34], while in pulmonary emphysema, IL-6 drives the activation of STAT3 in a different manner [35]. Together with IL-21 and IL-23, the IL-6 via latent STAT3 regulates the differentiation of Th17 cells and consequently increases the IL-17 production [36, 37]. The higher number of Th17 cells and IL-17 is involved in the development of inflammatory response and emphysema observed in animals chronically exposed to cigarette smoke [38]. In accordance, the present study showed, for the first time, elevated IL-17 concentration in BAL and in the serum of smoke-exposed mice, which can be associated to increased pulmonary STAT3 expression and activation. Of note, aerobic exercise reduced STAT3 expression and activation in the three lung compartments studied, an effect accompanied to reduced pulmonary and systemic inflammation, including a plethora of classical COPD proinflammatory cytokines (IL-1*β*, IL-6, CXCL1, IL-17, and TNF-*α*). However, further studies using a genetically engineered mouse or pharmacological approaches aiming to test a causal relation between the effects of aerobic exercise on STAT3 and inflammatory response in a model of COPD need to be performed.

In summary, our data not only confirms that aerobic exercise training was able to reduce pulmonary COPD-related airway inflammation and lung emphysema but showed for the first time that aerobic exercise also can inhibit systemic inflammation in a COPD model and that such effects may have the involvement of exercise-inhibit STAT3 activation.
